# Male Pelvic Squamous Cell Carcinoma of Unknown Primary Origin

**DOI:** 10.1155/2014/953698

**Published:** 2014-11-13

**Authors:** Lauren Chiec, Sadhna Verma, Ady Kendler, Nagla Abdel Karim

**Affiliations:** ^1^University of Cincinnati College of Medicine, Cincinnati, OH 45267, USA; ^2^Division of Radiology, University of Cincinnati College of Medicine, Cincinnati, OH 45267, USA; ^3^Division of Pathology, University of Cincinnati College of Medicine, Cincinnati, OH 45267, USA; ^4^The Vontz Center for Molecular Studies, 3125 Eden Avenue, RM 1312, ML 0562, Cincinnati, OH 45267, USA

## Abstract

Pelvic squamous cell carcinoma of unknown primary origin has been described in several case reports of female patients. However, there have been no published reports describing male patients with pelvic squamous cell cancer of unknown primary origin. Our case describes a 52-year-old man who presented with right buttock pain, rectal urgency, and constipation. His physical examination demonstrated tenderness to palpation around his gluteal folds. Computed tomography scan of his abdomen and pelvis demonstrated a large mass in his retroperitoneum. The mass was determined to be squamous cell carcinoma of unknown primary origin. Additionally, the patient had small nodules in his right lower lung lobe and right hepatic lobe. The patient was treated with concomitant chemoradiation, including cisplatin and intensity-modulated radiation therapy, followed by carboplatin and paclitaxel. The patient achieved partial remission, in which he remained one year after his presentation. Our case is consistent with the literature which suggests that squamous cell carcinoma of unknown primary origin occurring outside of the head and neck region may have a more favorable prognosis than other carcinomas of unknown primary origin. Further studies are necessary to determine the most appropriate work-up, diagnosis, and optimal treatment strategies.

## 1. Introduction

Carcinomas of unknown primary (CUP) origin account for 3–5% of all malignancies and can be defined as “a heterogeneous group of metastatic tumors for which a standardized diagnostic work-up fails to identify the site of origin at the time of diagnosis” [1]. Among CUPs, squamous cell carcinoma accounts for 5–10%, often presenting in the cervical, supraclavicular, and inguinal lymph nodes [2]. Metastatic squamous cell CUP occurring in the pelvic cavity is extremely rare, with several case reports described involving female patients. These cases include a patient presenting with iliac lymph node metastases and a patient with an incidental pelvic mass found upon work-up for dysfunctional uterine bleeding [3, 4]. There have been no published reports describing male patients with pelvic squamous cell CUP. We herein describe a case of metastatic squamous cell CUP occurring in the pelvic cavity of a 52-year-old male patient.

## 2. Case Presentation

A 52-year-old Caucasian male presented to the emergency department after several weeks of right buttock pain, rectal urgency, and constipation. He denied systemic symptoms and had a normal physical examination, including digital rectal examination. The patient was given polyethylene glycol and discharged. Upon follow-up with his primary care physician, he continued to complain of buttock pain (now bilateral and tender to palpation around his gluteal folds), along with alternating diarrhea and constipation, and new urinary frequency. His physical examination was again normal and it was recommended for him to obtain a computed tomography (CT) scan of his abdomen and pelvis to evaluate for any neoplasm.

Of note, the patient's past medical history was significant for excision of a penile lesion 14 months prior to his presentation. The lesion was a granular cell tumor with overlying pseudo epitheliomatous hyperplasia with no evidence of malignancy.

**Figure 1:**
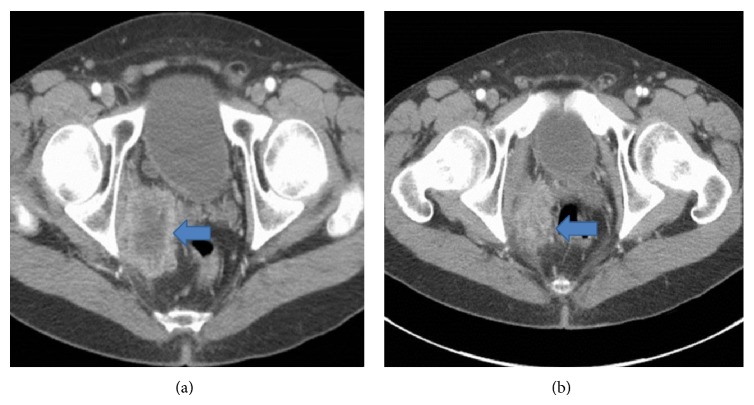
Contrast-enhanced CT scan of the pelvis demonstrates a 6.5 × 5.1 cm mass within the right pelvis (arrows), bordered by the right obturator internus, right seminal vesicles, and bladder diverticulum. The epicenter of the mass is centered at the internal iliac neurovascular bundle.

The CT scan was delayed for three months due to insurance issues. Subsequently when performed, it showed a large mass in his retroperitoneum. CT showed a 7 × 5 cm mass within the right pelvis, bordered by the right obturator internus, right seminal vesicles, and bladder diverticulum (Figures 1(a) and 1(b)). The epicenter of the mass was centered at the internal iliac neurovascular bundle. In addition to his pelvic mass, 5 and 3 mm nodules were discovered in his right lower lung lobe and right hepatic lobe, respectively.

**Figure 2:**
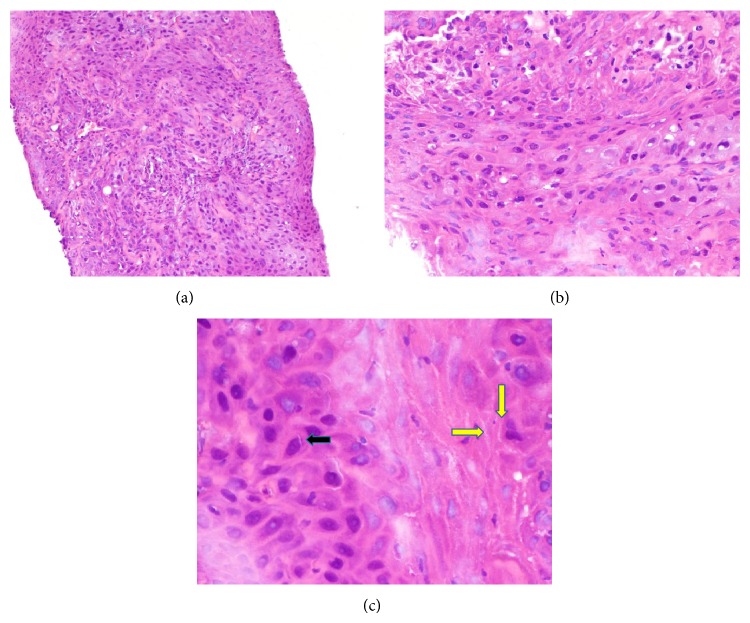
Hematoxylin and eosin (H&E) stain at 100x, 200x, and 400x power, respectively, demonstrates metastatic squamous cell carcinoma. At 400x power (c), intracellular bridges (yellow arrows) and eosinophilic, intracytoplasmic keratin (black arrow) are seen, indicating squamous differentiation.

**Figure 3:**
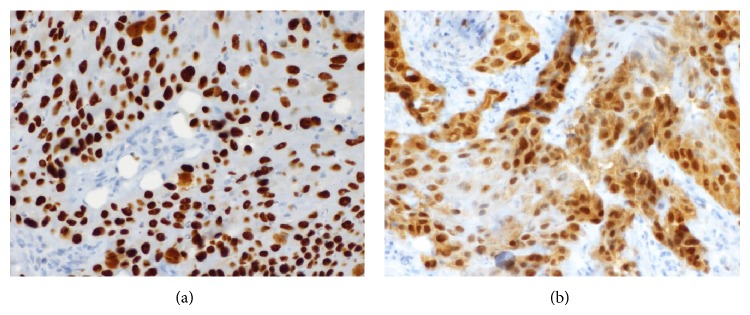
P63 immunostain shows positive nuclear staining in tumor cells ((a), (b)), as well as cytoplasmic staining (b), supporting the diagnosis of squamous cell carcinoma.

CT guided percutaneous biopsy showed a perirectal, invasive, moderately differentiated, keratinizing, squamous cell carcinoma (Figures 2(a), 2(b), and 2(c)). Immunostain was strongly positive for pankeratin and positive for CK5, CK6, and P16 (Figures 3(a) and 3(b)). It was negative for CK7 and PSA.

The patient began to have systemic symptoms of chills, diaphoresis, weight loss, decreased appetite, and fatigue, as well as increased hip, groin and abdominal pain, dysuria and frequency, and dizziness. Although anoscopy, cystoscopy, and CT chest were recommended for further work-up, these were not obtained given the patient's financial situation. Chemoradiation was started with 2 cycles of cisplatin (targeting penile, anal, lung, and bladder carcinoma) and intensity-modulated radiation therapy with 6000 centigray in 30 fractions. This was tolerated well.

Follow-up CT chest, abdomen, and pelvis after 2 cycles of cisplatin and concurrent radiation showed a decrease in the size of the right pelvic mass to 4.2 × 2 cm (35% decrease). At that time, the lesion was a well-defined fluid collection with no substantial soft-tissue component. The liver lesion was stable, and several pulmonary nodules were discovered. Several of the pulmonary nodules were stable from prior CT. No evidence of brain metastases were seen on brain MRI. The patient was incidentally found to have bilateral acute on chronic pulmonary emboli on CT chest and was admitted to the hospital and begun on low molecular weight heparin therapy. Systemic chemotherapy with weekly carboplatin and paclitaxel was begun soon after and the patient received two cycles of carboplatin and paclitaxel. This was tolerated well except for neutropenia which led to the delay of one cycle of his systemic therapy and continued grade 1 peripheral neuropathy.

Cystoscopy with biopsy was performed and showed urothelial mucosa with mild chronic inflammation, reactive epithelial changes, and focal squamous metaplasia. There was no evidence of malignancy.

**Figure 4:**
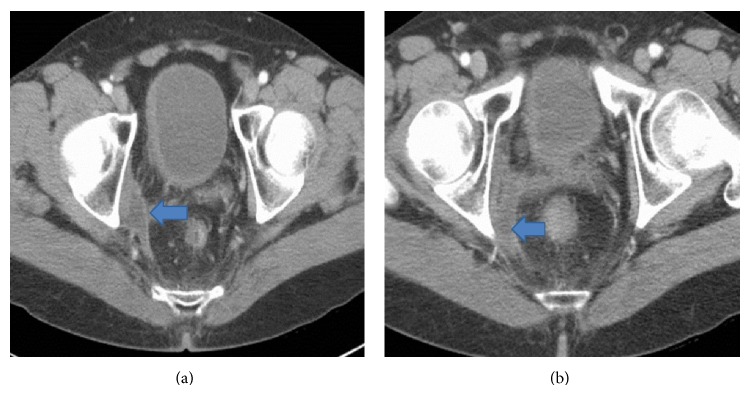
Contrast-enhanced CT scan of the pelvis demonstrates a 3.4 × 1.7 cm pelvic lesion (arrows) with no substantial soft-tissue component. The lesion is linear and elongated and appears to be comprised mainly of necrotic tissue, consistent with postradiation changes.

Follow-up CT chest, abdomen, and pelvis at 12 months after diagnosis (8 months after beginning treatment) showed a continued interval decrease in size of the pelvic lesion to 3.4 × 1.7 cm (Figures 4(a) and 4(b)). The patient has thus achieved a partial remission per RECIST 1.1 criteria [5], with an overall 47% decrease in the size of the lesion. In addition, the total response may be underestimated given that the residual mass had a linear and elongated shape and consisted of mainly necrotic tissue. Liver and lung lesions remain stable.

## 3. Discussion

In our report, we describe a 52-year-old male with pelvic squamous cell carcinoma of unknown primary origin, who achieved partial remission after platinum-based therapy in addition to paclitaxel. The uniqueness of this case is supported by the lack of published case reports describing male patients with this malignancy. Of the published literature regarding squamous cell CUP in the pelvic region, the patients described were all female, with gynecologic malignancies considered as possible etiologies.

One report describes a 47-year-old female with an incidental pelvic mass found during a work-up for dysfunctional uterine bleeding. It was classified as squamous cell CUP and the patient showed a complete response after radiotherapy and intensive long-term chemotherapy (six cycles of 5-FU, cisplatin and leucovorin; four cycles of etoposide, doxorubicin, and cisplatin; five cycles of ifosfamide, mesna, cisplatin, and etoposide). Although she had recurrence of her pelvic mass five years after completing treatment, her initial response was promising in demonstrating the effect of systemic chemotherapy on squamous cell CUP presenting in the pelvic cavity. The patient remained alive more than eight years after her diagnosis, suggesting the prognosis for this metastatic malignancy is more favorable than others [4].

Another report describes a 70-year-old female with persistent right leg edema, found to have squamous cell CUP metastatic to the iliac lymph nodes. She underwent surgical resection and radiation therapy and continued to show no recurrence after 17 months of follow-up. Her tumor was controlled with locoregional management; the role of systemic chemotherapy has not been evaluated in patients with CUP of the inguinal and or iliac lymph nodes [3].

In general, CUP has a poor prognosis, with a median survival time of less than six months and only about 15% of patients surviving after 1 year in population-based studies [6]. However, it appears that squamous cell carcinomas with limited anatomic distribution in nodal regions outside the head and neck region may be a prognostically favorable subset of CUP, with a median survival time of almost 2 years [7]. Our patient's clinical course is consistent with the literature in that he remains in partial remission one year after presentation. Given the rarity of squamous cell CUP, especially in the pelvic region, further studies are needed to fully evaluate the prognosis of this malignancy.

In our case, we were able to rule out many common primary malignancies, including prostate (immunostain PSA negative), urinary bladder (no evidence of malignancy on cystoscopy), and lung (immunostain p16 positive and CK7 negative). Immunostaining is not generally helpful in differentiating metastatic squamous cell CUP, aside from in favoring an extrapulmonary neoplasm if results are p16 positive [8]. P16 positivity is also seen in other primary malignancies associated with human papillomavirus infection, including squamous cell carcinomas of the oropharynx, cervix, and anus. Studies have shown that in some cases, a clone of cells in an otherwise benign epithelium may be determined as a source for metastases through microsatellite analysis before histopathology can identify definite dysplasia or malignancy [9]. In our patient, a primary anal neoplasm was unlikely as no anal lesions were noted on digital rectal examination. It is possible that targeted biopsies of common primary sites may be helpful in determining the origin of the metastases, although the rarity of this presentation makes the diagnostic approach challenging, and there are several obvious risks and financial limitations of further biopsies. Although there has been a case report of a patient presenting with pelvic squamous cell CUP which was later discovered to be primary esophageal carcinoma, this is rare and our patient has not shown any sign of esophageal neoplasm on CT chest studies [10].

Although our patient's clinical course and diagnostic work-up were consistent with a CUP, it is important to note that his initial staging investigations were incomplete, as the patient had significant financial constraints which limited his ability to undergo complete testing. He chose not to undergo anoscopy or sigmoidoscopy, and CT chest and cystoscopy were both performed after the patient had begun receiving systemic treatment. Had these investigations been performed at the time of diagnosis, the likelihood of discovering a primary site would have been much higher. Performing these investigations after treatment was begun could mean that the primary was no longer visible and therefore missed. In addition, although routine serum tumor markers are often considered in the initial diagnostic evaluation, these were not performed in our case. Routine tumor markers have been shown to be nonspecifically elevated in more than 50% of patients with CUP and have not been proven to have any diagnostic or prognostic significance at this time [11].

Based on the limited data from reported cases, it appears that squamous cell CUP, especially outside of the head and neck region, has a more favorable prognosis compared to other CUPs. It also appears that these malignancies may respond well to a combination of surgical resection, when feasible, local radiotherapy, and platinum-based systemic chemotherapy. Given the rare nature of pelvic squamous cell CUP, further reports and studies on these cases are necessary to better determine the ideal work-up for diagnosing primary tumors, optimal treatment strategies, and accurate prognostic data.

## 4. Conclusion

Squamous cell carcinoma of unknown primary origin is a rare malignancy, and reports of this neoplasm occurring in the pelvic cavity are extremely limited. Further studies are needed to determine ideal strategies for diagnosis, treatment, and prognostication.
